# No association between fertility desire and HIV infections among men and women: Findings from community-based studies before and after implementation of an early antiretroviral therapy (ART) initiation program in the rural district of North-western Tanzania

**DOI:** 10.12688/openresafrica.13432.1

**Published:** 2022-09-14

**Authors:** Denna Mkwashapi, Jim Todd, Michael Mahande, John Changalucha, Mark Urassa, Milly Marston, Jenny Renju

**Affiliations:** 1 Department of Sexual and Reproductive Health Research, Tanzania National Institute for Medical Research, Mwanza, Tanzania, Tanzania; 2 Department of Population Health, London School of Hygiene & Tropical Medicine, London, United Kingdom, UK; 3 Department of Epidemiology and Biostatistics, Kilimanjaro Christian Medical University College, Moshi, Tanzania, Tanzania

**Keywords:** Fertility, Fertility desire, WLWHI, HIV and ART

## Abstract

**Background:** Fertility is associated with the desire to have children. The impacts of HIV and antiretroviral therapy (ART) on fertility are well known, but their impacts on the desire for children are less well known in Tanzania. We used data from two studies carried out at different periods of ART coverage in rural Tanzania to explore the relationship between HIV infection and fertility desires in men and women.

**Methods:** We conducted secondary data analysis of the two community-based studies conducted in 2012 and 2017 in the Magu Health and Demographic system site, in Tanzania. Information on fertility desires, HIV status, and social–economic and demographic variables were analyzed. Fertility desire was defined as whether or not the participant wanted to bear a child in the next two years. The main analysis used log-binomial regression to assess the association between fertility desire and HIV infection.

**Results:** In the 2012 study, 43% (95% CI 40.7-45.3) of men and 33.3% (95% CI 31.8 - 35.0) women wanted another child in the next two years. In 2017 the percentage rose to 55.7% (95% CI 53.6 - 57.8) in men and 41.5% (95% CI 39.8 - 43.1) in women. Although fertility desire in men and women were higher in HIV uninfected compared to HIV infected, age-adjusted analysis did not show a statistical significance difference in both studies (2012: PR=1.02, 95%CI 0.835 - 1.174, p<0.915 and 2017: PR = 0.90 95%CI 0.743 - 1.084 p= 0.262).

**Discussion:** One-third of women and forty percent of men desired for fertility in 2012, while forty percent of women and nearly half of men desired for fertility in 2017. The data showed fertility desire, in 2012 and 2017 were not related to HIV infection in both periods of ART coverage.

## Introduction

Fertility desire in both men and women is associated with fertility ^1^. Fertility desire has been measured differently across Sub Saharan Africa (SSA) depending on the context of the question. It is described as the desire to stop childbearing ^2^, whereas in the other paper, it is described as the desire to bear a child, within a given time period, and usually in women ^3^. Fertility desire is of great interest to demographers and scientists as it forecasts future population fertility and the incidence of unwanted pregnancies.

Due to differences in measurements of fertility desire, fertility desire reports vary across SSA countries, ranging from 8% to 82% ^4^. In recent studies in SSA , it was reported that One-third of Malawian women ^5^, half of Ethiopian women ^6– 10^, and two-thirds of Ugandan women desired for one or more child in the future ^11^. In 2014, Ugandan study, reported a higher fertility desire in younger women and women with low social economic status ^12^, whilst another qualitative study in Zambia, showed a low fertility desire in women with reduced parity and in the family with bigger than expected family size ^13^.

HIV infection has been considered the major factors to influence fertility behaviour and fertility desire in women of reproductive age. In recent years, studies conducted in SSA had showed a lower fertility desire in women living with HIV (WLHIV) compared to HIV-uninfected women ^14– 17^. Yeatman and colleagues conducted a qualitative study and found that self-assessed likelihood of HIV infection reduced desire for fertility in women, while women who had a positive HIV test result reduced their fertility desire. WLHIV hides their fertility desire to be known to society because of the fear for mother-to-child transmission of HIV ^18^. Fertility desire in WLHIV is largely due to HIV associated stigma ^19^, while other women fear the physical health consequences of pregnancy and childbearing while living with HIV ^20^. However, some studies have shown that HIV infection does not diminish the desire to be pregnant and bear a child ^21^. Therefore the impact of HIV infection on fertility is well known, but the impact of HIV infection on fertility desire is equivocal.

Antiretroviral therapy (ART) availability has reduced the risk of vertical HIV transmission and improved the wellbeing of the WLHIV. However, ART usage may motivate WLHIV to have more children despite HIV positive diagnosis. Studies in SSA have shown that ART usage increased fertility desires ^19– 22^. ART had been said to resume the quality of life for people living with HIV (PLHIV) and enhance their desire for children ^23– 26^. In Tanzania, up to 2012, ART initiation was based on diminishing immune cells markers (CD4 counts), with only 14% of PLHIV receiving ART in 2012 ^28^. In 2013, the Prevention of Mother to Child HIV Transmission (PMTCT) program ensured lifelong free ART was given to all pregnant women diagnosed with HIV regardless of their disease stage, viral or CD4 cell counts ^29^. In 2016, the universal HIV test and Treat (UTT) policy provided ART to all HIV-infected individuals regardless of their immune status ^30, 31^ The impact of ART and/or HIV on population fertility rate and desire may be more pronounced in countries with high HIV prevalence, HIV testing rate, and ART coverage ^32^.

In 2018, Tanzanian data shows that, for women aged 15 years and above, 82% have tested for HIV in the past year and 82% of those found positive have initiated ART ^33^. It is unclear whether the increased ART availability due to earlier ART initiation and PMTCT option B plus has impacted on the fertility desire in WLHIV. In this analysis, we used data from two repeated community-based studies carried out in 2012 and 2017 to explore the levels of fertility desires and its association with HIV infection in men and women. 

## Methods

### Ethical consideration

All participants who contributed data for analysis provided written informed consent for study participation and publication of the results. Parents or any care giver consented on behalf of study participants who were aged less than 18 years. Ethical approvals were obtained from the Lake Zone Institutional Review Board (MR/53/100/513), the Ethical Review Committee of Kilimanjaro Christian Medical College of the Tumaini University of Tanzania (certificate number 2440) and from the London School of Hygiene and Tropical Medicine. (LSHTM Ethics Ref: 8623). 

### Study setting and design

The 2012 and 2017 studies were conducted in a health and demographic sentinel surveillance population in the Magu District of north-western Tanzania (Magu HDSS). Magu HDSS has a population of 45,000 with the majority, dwelling in the rural areas, belonging to Sukuma ethnic group and of the Christian religion. The main economic activities are small-scale farming, livestock keeping, and petty businesses involving agricultural and livestock products ^34^. The HIV Serological surveillance system (sero survey) is nested within the Magu HDSS, with details described elsewhere ^34, 35^. The codebook for 2012 and 2017 sero-survey datasets, user guide for 2012 and 2017 sero-survey datasets and the information sheet and consent form for sero-survey can be found in Extended data ^42^.

After consent, sero survey participants responded to structured face-to-face interview, collecting quantitative information on; demographics, fertility desire, child-bearing and family planning.

### Data and variables

Primary outcome variable was the fertility desire defined as the desire to bear one or more child in the next two years. Both women and men answered the questions “Would you like to have an (other) child?”, “How soon would you like your next child to be born?” and “How many more children would you like to have?” Fertility desire variable was binary and was defined as a proportion with fertility desire (desire to bear one or more children in the next two years) against all aged 15–49 year’s age. For comparison purpose, we restrict the age of men to be between 15–49 years.

The exposure of interest was HIV infection, and was abstracted from the stored HIV test results. HIV testing was done in serological surveillance system and measured through a standardized Tanzanian protocol for HIV testing ^36^. Demographic details of the participants were collected through the standardized serological surveillance questionnaire. The questionnaire collected information on age, marital status, education level, residence, occupation, religion, ethnicity, and lifestyles which included alcohol drinking habit and Cigarette smoking. We also had variables to represent the past obstetric history of the women including the number of previous pregnancies. Data entry and management were done using the Census and Survey Processing System software (CSPro) version 6.3.

### Statistical analysis

The descriptive analysis reported the prevalence of fertility desire in men and women with 95% confidence intervals (95% CI) in each study, both overall and by exposure variables. We computed the association between fertility desire and HIV infection by calculating crude and adjusted estimates of prevalence ratio (PR) with 95% CI by using the log-binomial regression. All analyses were done separately for 2012 and 2017 studies. Analysis was done using STATA, version 16.1 (StataCorp, College Station, TX) statistical package.

## Results

There were 5221 and 5730 participants aged 15–49 years in 2012 and 2017 respectively. In the 2012 study, there were 3361 women (64.4%) of whom 434 (12.6%) were WLHIV and 1860 men (35.6%) of whom 156 (8.4%) were men living with HIV. In 2017 there were 3560 women (62.1%) and 2164 men (37.9%), of whom 257 (7.2%) women and 101 (4.7%) men were living with HIV. Details on the characteristics of men and women participants in each study are shown in Table 1.

**Table 1.** Characteristics of men and women who participated in the 2012 and 2017 studies.

		2012 Study				2017 Study			
		Women		Men		Women		Men	
Variable	Characteristics	Number	Percent	Number	Percent	Number	Percent	Number	Percent
Fertility desire									
	No	2239	66.6	1060	57.0	2087	58.5	959	44.3
	Yes	1,122	33.4	800	43.0	1479	41.5	1205	55.7
HIV status									
	HIV – Positive	434	12.6	156	8.4	257	7.2	101	4.7
	HIV – Negative	3,013	87.4	1707	91.6	3318	92.8	2051	95.3
Age in groups									
	15 – 24	1357	39.3	954	51.2	1524	42.3	1199	55.2
	25 – 34	1044	30.3	401	21.5	1005	27.9	402	18.5
	35 – 49	1049	30.4	509	27.3	1072	29.8	571	26.3
Education level									
	None	852	24.7	236	12.8	833	23.1	214	9.9
	Primary (1–4)	190	5.5	138	7.5	149	4.1	157	7.2
	Primary (5–7)	1938	56.2	936	50.6	1838	51.0	989	45.6
	Secondary and Tertiary	467	13.6	538	29.1	781	21.7	810	37.3
Place of Residence									
	Rural	2222	64.4	1381	74.1	2036	56.5	1388	63.9
	Urban	1228	35.6	483	25.9	1565	43.5	784	36.1
Alcohol taking									
	No	3344	96.9	1581	84.8	3435	95.9	1796	83.0
	Yes	106	3.1	283	15.2	146	4.1	368	17.0
Cigarette Smoking									
	No	3431	99.4	1642	88.1	3556	99.3	1908	88.2
	Yes	19	0.6	222	11.9	25	7.0	256	11.8
Religion									
	Christians	3190	92.5	1414	75.9	3264	91.1	1771	81.8
	Non Christians	260	7.5	450	24.1	317	8.9	393	18.2
Ethnicity									
	Sukuma	3228	93.6	1777	95.3	3283	91.7	2024	93.5
	Non Sukuma	222	6.4	87	4.7	298	8.3	140	6.5
Earning money									
	No	1072	31.1	648	34.8	1242	34.7	723	33.4
	Yes	2378	68.9	1216	65.2	2339	65.3	1441	65.6
Ever been Pregnant									
	No	743	21.5			894	25.0		
	Yes	2706	78.5			2687	75.0		

### Fertility desire in men and women

**Figure 1.**
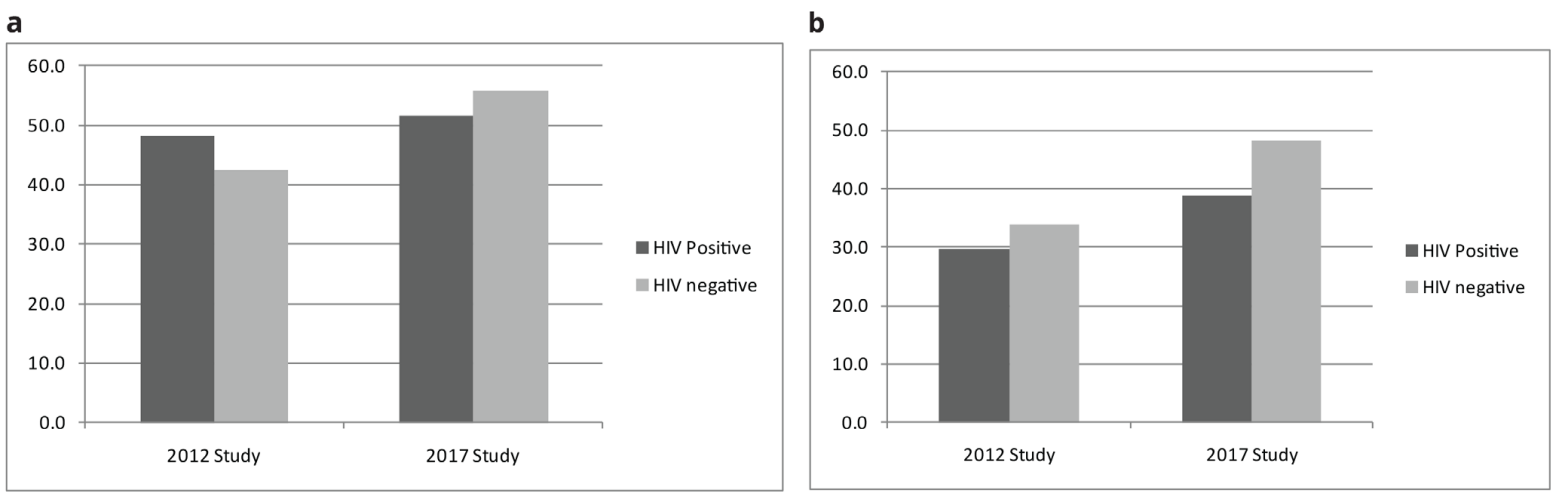
**a.** Fertility desire by HIV infection by year of the Study - men. **b.** Fertility desire by HIV infection by year of the Study - women.

**Figure 2.**
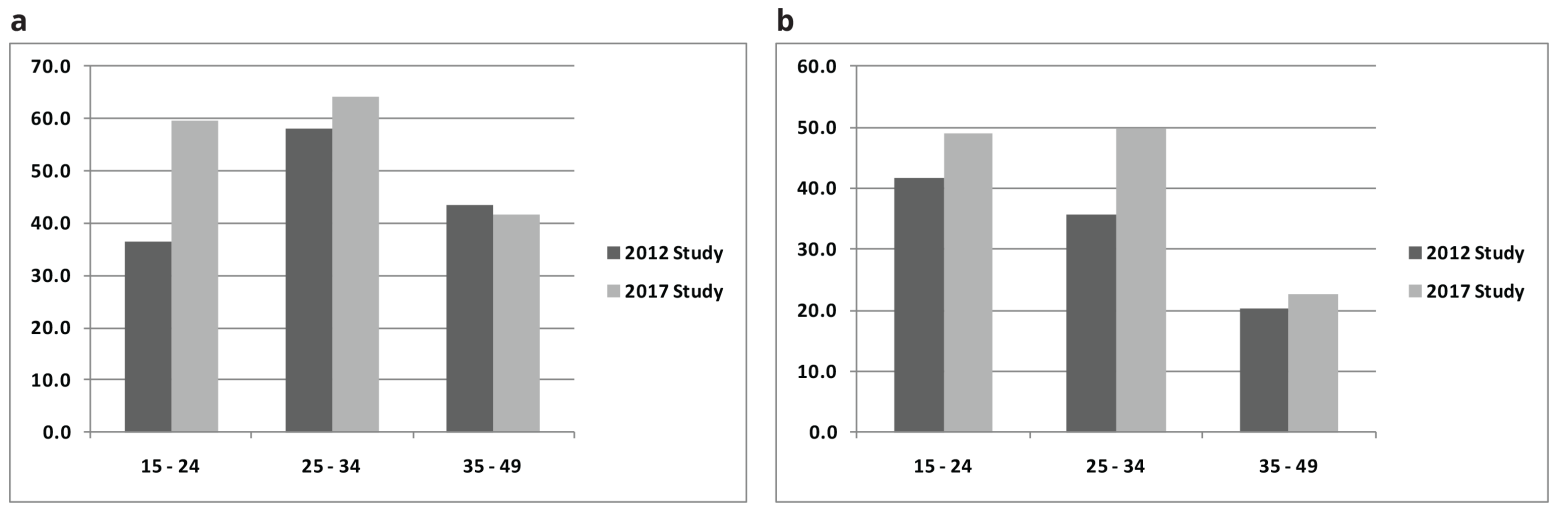
**a.** Fertility desire by age by years of the Study - Men. **b.** Fertility desire by age by years of the Study - Women.

In the 2012 study, the overall percentage of the desire to have one or more child in the next two years in men and women was 43.0% (95% CI 40.7 - 45.3) and 33.3% (95% CI 31.8 - 35.0) respectively, while in 2017, the percentage desire for fertility rose to 55.7% (95% CI 53.6 - 57.8) in men and 41.5% (95% CI 39.8 - 43.1) in women. In both studies, fertility desires in men and women living with HIV were relatively lower compared to the fertility desires in HIV uninfected men and women. Fertility desire for women living with HIV was 29.6% (95% CI 25.3 - 34.3) in the 2012 and 38.9% (95% CI 95% 32.7 - 44.9) in 2017. Whilst fertility desire in men living with HIV was 48.1% (95% CI 40.0 - 56.2) in the 2012 survey and 51.5% (95% CI 41.3 - 61.5) in the 2017 survey ( Table 2). The level of fertility desire, with or without HIV infection, showed that the desires in men were almost always higher than that of women in both studies ( Figure 1). Overall, the fertility desires tended to decrease with increasing age notably with a higher fertility desire in men than in women in both surveys ( Figure 2).

**Table 2.** Women and men fertility desire and 95% Confidence Intervals in the 2012 and 2017 studies.

		2012 Study				2017 Study			
Variable	Characteristics	Women's fertility desire in % (95%CI)		Men's fertility desire in % (95%CI)		Women's fertility desire in % (95%CI)		Men's fertility desire in % (95% CI)	
All (15-49)		33.4	31.8 - 35.0	43.0	40.7 - 45.3	41.5	39.8 - 43.1	55.7	53.6 - 57.8
HIV status									
	HIV – Positive	29.6	25.3 - 34.3	48.1	40.0 - 56.2	38.7	32.7 - 44.9	51.5	41.3 - 61.5
	HIV – Negative	33.9	32.2 - 35.7	42.5	40.2 - 44.9	41.7	40.0 - 43.4	55.8	53.6 - 57.9
Age in groups									
	15 – 24	41.8	39.1 - 44.5	36.3	33.3 - 39.5	49.0	46.5 - 51.6	59.5	56.6 - 62.3
	25 – 34	35.8	32.9 - 38.8	58.1	53.1 - 62.9	49.8	46.6 - 52.9	64.3	59.3 - 69.0
	35 – 49	20.4	18.0 - 23.0	43.6	39.3 - 48.0	22.5	20.0 - 25.2	41.6	37.6 - 45.8
Education level									
	None	32.7	29.5 - 36.1	56.4	49.8 - 62.8	39.6	36.2 - 43.1	53.9	46.8 - 60.8
	Primary (1–4)	33.9	27.2 - 41.1	60.6	51.9 - 68.9	38.9	31.1 - 47.2	63.7	55.7 - 71.2
	Primary (5–7)	35.3	33.2 - 37.5	43.0	39.8 - 46.3	42.6	40.3 - 44.9	54.4	51.2 - 57.5
	Secondary and Tertiary	26.2	22.2 - 30.5	33.0	29.0 - 37.1	41.2	37.8 - 44.8	56.2	52.8 - 59.7
Place of Residence									
	Rural	33.1	31.1 - 35.1	44.2	41.5 - 46.8	45.8	43.6 - 48.0	60.2	57.5 - 62.8
	Urban	33.9	31.2 - 36.7	39.8	35.4 - 44.3	35.8	33.4 - 38.2	47.8	44.2 - 51.3
Alcohol taking									
	No	33.5	31.9 - 35.2	41.3	38.9 - 43.8	41.9	40.2 - 43.6	56.8	54.5 - 59.1
	Yes	28.8	20.4 - 38.6	52.3	46.3 - 58.2	31.7	24.2 - 39.9	50.3	45.0 - 55.5
Cigarette Smoking									
	No	33.4	31.8 - 35.1	42.0	39.6 - 44.4	41.4	39.8 - 43.1	56.4	54.2 - 58.7
	Yes	22.2	6.4 - 47.4	50.5	43.6 - 57.2	48.0	27.8 - 68.7	50.0	43.7 - 56.3
Religion									
	Christians	33.5	31.8 - 35.2	39.9	37.3 - 42.5	41.5	39.8 - 43.2	55.3	52.9 - 57.7
	Non Christians	32.4	26.7 - 38.5	52.8	48.1 - 57.5	41.1	35.6 - 46.7	57.3	52.2 - 62.2
Ethnicity									
	Sukuma	33.4	31.7 - 35.1	43.1	40.8 - 45.4	41.6	39.9 - 43.3	56.1	53.9 - 58.3
	Non Sukuma	33.4	27.2 - 40.0	41.4	30.9 - 52.4	39.7	34.1 - 45.5	50.0	41.4 - 58.6
Earning money									
	No	32.0	29.1 - 34.9	25.9	22.6 - 29.5	38.1	35.4 - 40.9	55.5	51.8 - 59.1
	Yes	34.0	32.1 - 36.0	52.1	49.3 - 55.0	43.3	41.2 - 45.3	55.8	53.2 - 58.4
Ever been Pregnant									
	No	39.3	35.7 - 42.9			41.5	38.2 - 44.8		
	Yes	31.7	29.9 - 33.6			41.5	39.6 - 43.4		

### Association between HIV infection and women’s fertility desire

Although women’s fertility desire was higher in HIV uninfected women compared to WLHIV in 2012 and 2017, age-adjusted analysis, did not show significant statistical association in 2012 (PR=1.02, 95CI 0.860 - 1.187, p = 0.847) and 2017 (PR= 0.99, 95%CI 0.835 - 1.174 p= 0.915). Details of result are highlighted in Table 3. In the multivariable analysis model using log-binomial regression, (adjusted for age, education level, place of residence, earning money for the living and contraceptive use), our data did not show significant statistical association in 2012 (Adjusted PR= 1.02, 95% CI 0.852 - 1.227, p= 0.0.814) and 2017 (Adjusted PR= 0.91, 95% CI 0.790 - 1.057, p= 0.225).

**Table 3.** Crude and Age-Adjusted risk ratio with 95% Confidence Intervals for factors associated with women's and men's fertility desire in the 2012 study.

	Women						Men					
	Crude analysis			Age- adjusted analysis			Crude analysis			Age- adjusted analysis		
Category	Risk Ratio	95% Confidence intervals	P value	Risk Ratio	95% Confidence intervals	P value	Risk Ratio	95% Confidence intervals	P value	Risk Ratio	95% Confidence intervals	P value
HIV status												
HIV – Positive	Ref			ref			ref		ref			
HIV – Negative	1.14	0.978 - 1.338	0.092	1.02	0.86 - 1.187	0.847	0.88	0.744 - 1.050	0.162	0.99	0.835 - 1.174	0.915
Age in groups												
15 – 24	Ref						ref					
25 – 34	0.86	0.772 - 0.951	0.004				1.60	1.421 - 1.801	<0.001			
35 – 49	0.49	0.427 - 0.561	<0.001				1.20	1.055 - 1.367	0.006			
Education level												
None												
Primary (1–4)	1.03	0.829 - 1.292	0.763	0.98	0.795 - 1.217	0.882	1.08	0.902 - 1.281	0.419	1.08	0.914 - 1.274	0.367
Primary (5–7)	1.08	0.962 - 1.212	0.192	0.95	0.848 - 1.064	0.374	0.76	0.667 - 0.873	<0.001	0.80	0.670 - 0.910	0.001
Secondary and Tertiary	0.80	0.666 - 0.960	0.017	0.56	0.462 - 0.674	<0.01	0.58	0.496 - 0.690	<0.001	0.65	0.550 - 0.772	<0.001
Place of Residence												
Rural												
Urban	1.02	0.929 - 1.133	0.607	1.07	0.974 - 1.181	0.157	0.90	0.794 - 1.020	0.099	0.90	0.799 - 1.019	0.096
Alcohol taking												
No												
Yes	0.86	0.34 - 1.168	0.335	1.03	0.761 - 1.386	0.885	1.26	1.115 - 1.434	<0.001	1.11	0.975 - 1.270	0.112
Religion												
Christians												
Non Christians	0.97	0.806 - 1.164	0.736	1.02	0.857 - 1.225	0.786	1.32	1.186 - 1.474	<0.001	1.27	1.146 - 1.415	<0.001
Ethnicity												
Sukuma												
Non Sukuma	1.03	0.826 - 1.219	0.001	1.01	0.827 - 1.210	0.993	0.96	0.743 - 1.240	0.756	0.95	0.744 - 1.223	0.713
Earning money												
No												
Yes	1.06	0.958 - 1.182	0.244	1.38	1.236 - 1.540	<0.01	2.01	1.747 - 2.320	<0.001	2.13	1.815 - 2.489	<0.001
Ever been Pregnant												
No												
Yes	2.01	1.747 - 2.231	<0.001	1.14	1.002 - 1.293	0.045						

Women who were earning money for their family were found to have an increased desire to bear one or more children in the next two years in both 2012 (adjusted PR= 1.98, 95% CI 1.668 - 2.344, p<0.001) and in 2017 (adjusted PR= 1.43, 95% CI 1.297 - 1.571, p<0.001) when compared to those who were not earning money for their family. In the 2012 and 2017, fertility desire was lower in women who were using modern contraceptives than women who had never used modern family planning methods in both 2012 (adjusted PR= 0.68, 95% CI 0.527 - 0.885, p=0.004) and 2017(adjusted PR= 0.89, 95% CI 0.807 - 0.977, p=0.015). Details of result are presented in Table 5.

### Association between HIV infection and men’s fertility desire

Although fertility desire in men was higher in HIV uninfected compared to HIV infected in both data, age-adjusted analysis of did not show statistical significance differences in men’s fertility desire by HIV status. (2012: PR=1.02, 95%CI 0.835 - 1.174, p<0.915 and 2017: PR = 0.90 95%CI 0.743 - 1.084 p= 0.262). Details of result are highlighted in Table 4. In both studies, men’s desire to have one or more children in the next two years decreased in those who was living in urban settlements (2012: PR= 0.81, 95%CI 0.741 - 0.867, P<0.001, 2017: PR= 0.79, 95%CI 0.726 - 0.858, P<0.0001) compared to rural villages. Details of result are available in Table 5.

**Table 4.** Crude and Age-Adjusted risk ratio with 95% Confidence Intervals for factors associated with women's and men's fertility desire in the 2017 Study.

	Women						Men					
	Crude analysis			Age-adjusted analysis			Crude analysis			Age-adjusted analysis		
Category	Risk Ratio	95% Confidence intervals	P value	Risk Ratio	95% Confidence ntervals	P value	Risk Ratio	95% Confidence intervals	P value	Risk Ratio	95% Confidence intervals	P value
HIV status												
HIV - Positive	Ref			ref			ref			ref		
HIV - Negative	1.07	0.920 - 1.265	0.352	0.89	0.769 - 1.040	0.148	1.08	0.893 - 1.315	0.412	0.90	0.743 - 1.084	0.262
Age in groups												
15 – 24	Ref						ref					
25 – 34	1.01	0.937 - 1.100	0.711				1.08	0.990 - 1.177	0.083			
35 – 49	0.46	0.406 - 0.512	<0.001				0.70	0.628 - 0.780	<0.001			
Education level												
None	Ref			ref			ref			ref		
Primary (1–4)	0.98	0.790 - 1.223	0.877	0.94	0.762 - 1.150	0.531	1.18	0.994 - 1.405	0.058	1.16	0.981 - 1.364	0.083
Primary (5–7)	1.08	0.972 - 1.189	0.154	0.98	0.892 - 1.082	0.72	1.01	0.879 - 1.160	0.893	1.01	0.888 - 1.159	0.833
Secondary and Tertiary	1.04	0.923 - 1.173	0.509	0.77	0.679 - 0.874	<0.01	1.04	0.908 - 1.201	0.541	0.95	0.824 - 1.086	0.436
Place of Residence												
Rural	Ref			ref			ref			ref		
Urban	0.78	0.720 - 0.848	<0.001	0.81	0.741 - 0.867	<0.01	0.79	0.729 - 0.864	<0.001	0.79	0.726 - 0.858	<0.001
Alcohol taking												
No												
Yes	0.75	0.594 - 0.964	0.024	0.91	0.720 - 1.144	0.412	0.89	0.794 - 0.987	0.029	1.01	0.894 - 1.128	0.938
Religion												
Christians	Ref			ref			ref			ref		
Non Christians	0.99	0.861 - 1.136	0.883	1.04	0.908 - 1.181	0.604	1.03	0.940 - 1.138	0.483	1.06	0.964 - 1.159	0.237
Ethnicity												
Sukuma	Ref			ref			ref			ref		
Non Sukuma	0.95	0.825 - 1.104	0.53	1.06	0.964 - 1.159	0.237				0.89	0.752 - 1.057	0.186
Earning money												
No	Ref			ref			ref			ref		
Yes	1.14	1.044 - 1.237	0.003	1.04	0.906 - 1.181	0.604	1.01	0.929 - 1.089	0.884	1.06	0.964 - 1.159	0.237
Ever been Pregnant												
No	Ref			ref								
Yes	0.99	0.914 - 1.094	0.997	1.39	1.257 - 1.537	<0.01						

**Table 5.** Adjusted Risk ratios for the factors associated with women's fertility desire to bear more children in the 2012 and 2017 Studies.

Variables	Category	2012 Study			2017 Study		
		Adjusted Risk Ratio	95% Confidence intervals	P value	Adjusted Risk Ratio	95% Confidence intervals	P value
HIV status							
	HIV – Positive	ref					
	HIV – Negative	1.02	0.852 - 1.227	0.814	0.91	0.790 - 1.057	0.225
Age in groups							
	15 – 24	ref					
	25 – 34	1.13	0.989 - 1.291	0.073	0.87	0.800 - 0.954	0.003
	35 – 49	0.86	0.743 - 0.999	0.048	0.38	0.335 - 0.435	<0.001
Education level							
	None	ref					
	Primary (1–4)	1.15	0.956 - 1.378	0.138	0.99	0.810 - 1.212	0.93
	Primary (5–7)	0.89	0.763 - 1.027	0.109	1.01	0.927 - 1.119	0.701
	Secondary and Tertiary	0.84	0.702 - 1.013	0.068	0.93	0.814 - 1.055	0.252
Place of Residence							
	Rural	ref					
	Urban	0.98	0.866 - 1.109	0.752	0.87	0.802 - 0.943	0.001
Earning money							
	No	ref					
	Yes	1.98	1.668 - 2.344	<0.001	1.43	1.297 - 1.571	<0.001
Family Planning							
	Never used	ref					
	Ever and currently using	0.68	0.527 - 0.885	0.004	0.89	0.807 - 0.977	0.015

## Discussion

In our study, one-third of women and forty percent of men desired to have one or more children in the next two years in 2012, while forty percent of women and nearly half of men desired to have one or more children in the next two year in 2017. In 2017, HIV infection did not have an impact on fertility desire in either men or women, and this did not differ by different periods of ART provision. Increased fertility desire was associated in women who earned money for the family, living in rural areas and lower educational attainment. Decreased fertility desire was associated with increasing age and men generally tended to have a higher fertility than women.

The data from 2012 relate to a time when ART was only being used by a few people. By 2017 increased access to ART through earlier ART initiation and PMTCT option B plus in pregnant women had been rolled out. However, the impact of HIV on fertility desire did not change over this time, indicating that the population effect during the period of widespread availability of ART only increased fertility desire in men and women, but may not the observed gap in fertility desire in positives and negatives men and women.

Our findings support others on showing the prevalence of women’s fertility desire in SSA ^7, 8^. The systematic review conducted by Martins and his colleagues in 2019, showed the prevalence of fertility desire in SSA to vary greatly ^4^, with some of the findings being consistent with ours ^9, 37^. However, some results are conflicting with ours by reporting a lower prevalence of women’s fertility desire ^38, 39^ and extremely higher prevalence than ours ^40^. Due to great variation on fertility desire estimates, researchers have suggested two main sources of variations in measuring fertility desires in SSA: internal and external sources of variations. Internal sources of variation included characteristics and sizes of study samples, data collection methods, definition of fertility desires and/or its assessment methods. External sauces of variation included social demographic, economic and cultural characteristics of a locality or segment of the population whether it is richly or poorly resourced country ^41^.

Compared to HIV-uninfected women, there was no evidence of higher fertility desire in WLHIV in the period of earlier ART initiation program implementation (2017). In the Ugandan study, Lindsay and colleges supported our results by reporting no statistical significant difference on fertility desire among ART/PMTCT service users and non-users (adj. PRR: 0.84, CI: 0.62-1.14) ^22^. Although the findings may not be comparable with our findings, the two studies differ in two main aspects 1) Our study did not have individual level data on ART and 2) our study reported on HIV sero-positivity alone with no information on whether the participants knew their HIV status. Ugandan study compared individual level ART data and possibly, participants were aware of their HIV status.

In our study, the following factors were found to increase women’s fertility desire: being in a position to earn money for the family, and history of previous pregnancy. However, we found a higher desire for fertility in women who attended secondary and tertiary education in the 2012 but disappeared later in the 2017. Among users of modern contraceptives, fertility desire was decreasing with increasing age. Several studies have reported a range of factors that explain changes in fertility desire, and some of their results correlated with ours ^7– 9^.

Our study's strengths lie in the adequate sample size to measure the change in fertility desire and reliable HIV sero-status results. Among study weaknesses - absence of a qualitative component to adequately and reasonably measure fertility desire: unavailability of individual-level ART data, fecundity for men and women and finally inability to look into the data longitudinally.

## Conclusion

We have reported a percentage of fertility desire in WLHIV in the period before and after earlier ART initiation program implementation in rural district of North western Tanzania. There was no evidence to suggest the difference in fertility desire between WLHIV and HIV-uninfected, over two difference phases of ART availability. The knowledge on factors associated with changes in fertility desire will be used in developing patient-centred reproductive health care in Tanzania. The healthcare services will include interventions against unplanned pregnancies for WLHIV and HIV-uninfected women and increased FP promotion campaigns in men and women.

## Data availability

### Underlying data

The data that support findings of this study cannot be shared publicly, but will be available upon request and following approval by The Medical Research Coordinating Committee (MRCC) of the National Institute for Medical Research (NIMR) in Tanzania. MRCC demand that all data collected within Tanzania may not be transferred or shared without their permission and before the signing of a data transfer agreement as the only criterion to access the data, which in line with the Government data protection policy.

For Researchers who wish to meet the above criteria for access to the data they should use the contact details below to request the data:

The Secretariat

Medical Research Coordinating Committee (MRCC),

National Institute for Medical Research,

2448, Barrack Obama Road, P O Box 9653

Dar es Salaam

Tanzania

E-mail: ethics@nimr.or.tz

The codebook for 2012 and 2017 sero-survey datasets, user guide for 2012 and 2017 sero-survey datasets and the information sheet and consent form for sero-survey can be found in Extended data ^42^.

### Extended data

LSHTM Data Compass: Data for: “Fertility desire for men and women: Magu Health and Demographic surveillance system”. https://doi.org/10.17037/DATA.00002883 ^42^.

This project contains the following extended data:

a) Fertility_desire_dataset_codebook.html (Codebook for 2012 and 2017 sero-survey datasets).
b) 2883_Userguide.html (User guide for 2012 and 2017 sero-survey datasets) and
c) Sero-survey_consent.pdf (Information sheet and consent form for sero-survey).

Data are available under the terms of the Creative Commons Attribution 4.0 International license (CC-BY 4.0).
