# Supplementary material for: No association between fertility desire and HIV infections among men and women: Findings from community-based studies before and after implementation of an early antiretroviral therapy (ART) initiation program in the rural district of North-western Tanzania
Source: Open Res Afr. 2022 Sep 14;5:30. [Version 1] doi: 10.12688/openresafrica.13432.1 (PMC10432893; doi:10.12688/openresafrica.13432.1)
Supplement: Peer review history

# Peer review: Reviewer response for version 1

**DOI:** 10.21956/openresafrica.14580.r30036

**Published:** 2023-08-16

**Authors:** Laura Tarancón-Díez

**Affiliations:** 1 Immunology Section, Laboratorio Inmuno-Biología Molecular (LIBM), Hospital General Universitario Gregorio Marañón (HGUGM), Instituto de Investigación Sanitaria Gregorio Marañón (IiSGM), Madrid, Spain; 2 Centro de Investigación Biomédica en Red Bioingeniería, Biomateriales y Nanotecnología (CIBER-BBN), Madrid, Spain

In the study setting and design section, information should include also qualitative information not only quantitative. For example, the response for the question “Would you like to have an (other) child?”, the response yes or no is qualitative.

 It is unclear why the age is restricted only for men? What about women? It should be interesting to indicate the age range for women and men as inclusion criteria, the range cannot necessarily be the same in both cases.

 In table 1 and table 2, the information of dichotomic variables is redundant. Indicate for fertility desire only the number and percentage of “yes” and in the case of HIV status, only the prevalence of HIV-positive. The same for alcohol taking, cigarette smoking earning money and even been pregnant. These changes will make the tables smaller and easy to understand. Indicate in a first top row the total number of participants per group and sex.

 In the conclusion section, FP abbreviation was not defined before.

English editing:

- One-third – one-third.
- Desired for one or more child – desire one or more child in the future.
- Bigger than expected family size – bigger family size than expected.
- However, ART usage may motivate WLHIV to have more children despite HIV positive diagnosis – Therefore, ART usage may…

Is the work clearly and accurately presented and does it cite the current literature?

Yes

If applicable, is the statistical analysis and its interpretation appropriate?

Yes

Are all the source data underlying the results available to ensure full reproducibility?

Yes

Is the study design appropriate and is the work technically sound?

Yes

Are the conclusions drawn adequately supported by the results?

Yes

Are sufficient details of methods and analysis provided to allow replication by others?

Yes

Reviewer Expertise:

Immunology, viral infections, inflammation, pediatric infectious diseases

I confirm that I have read this submission and believe that I have an appropriate level of expertise to confirm that it is of an acceptable scientific standard.

# Peer review: Reviewer response for version 1

**DOI:** 10.21956/openresafrica.14580.r30037

**Published:** 2023-08-16

**Authors:** Vincent Tukei

**Affiliations:** 1 EGPAF, Maseru, Lesotho

Denna Mkwashapi *et al. *have presented findings of a study designed to explore relationship between HIV infection and fertility desires in men and women. The study is based on secondary data analysis from 2 previous studies carried out in 2012 and 2017. The manuscript is generally well written; however, the authors may wish to attend to the following: 

1. There are a few grammatical errors in the Introduction section of the manuscript which may make it difficult for readers to understand some of the sentences. As an example, the first sentence of paragraph 3 may need some editing. 
2. Methods>Ethical Considerations: The authors state that "parents or any caregivers consented on behalf of study participants aged less than 18 years." Did any of these participants (<18 years) provide assent? More detail on the assent/consent process would bring clarity to this section? 
  "Any caregiver"- did this include boyfriends/girlfriends and husbands? 
3. Provide a brief summary in the methods to describe how participants were selected for enrolment to the 2012 & 2017 studies.
4. Results & Discussion: Notably absent in your data is the ART status of HIV-positive participants. How many of the 2012 participants were on ART at the time of the study? It is likely that all HIV positive participants in the 2017 study were on ART, and probably only a fraction of the HIV-positive participants were on ART in 2012. How did you handle this in your comparison of fertility desires? 

Is the work clearly and accurately presented and does it cite the current literature?

Yes

If applicable, is the statistical analysis and its interpretation appropriate?

I cannot comment. A qualified statistician is required.

Are all the source data underlying the results available to ensure full reproducibility?

Partly

Is the study design appropriate and is the work technically sound?

Yes

Are the conclusions drawn adequately supported by the results?

Yes

Are sufficient details of methods and analysis provided to allow replication by others?

Partly

Reviewer Expertise:

HIV

I confirm that I have read this submission and believe that I have an appropriate level of expertise to confirm that it is of an acceptable scientific standard, however I have significant reservations, as outlined above.

# Peer review: Reviewer response for version 1

**DOI:** 10.21956/openresafrica.14580.r29313

**Published:** 2022-10-11

**Authors:** Takele Menna Adilo

**Affiliations:** 1 Department of Public Health, St Paul's Hospital Millennium Medical College, Addis Ababa, Ethiopia

**General comment:** The manuscript seems technically sound and addresses a timely title.

**Specific comments:**

 Although it can be said that the work has academic merit, the type of epidemiological study design used is not clearly stated.

 I think there are contradictory concepts in the methods section. For instance, as stated in the abstract the authors conducted secondary data analysis of the two community-based studies of 2012 and 2017 in the Magu Health and Demographic system site, in Tanzania. To the contrary, in the methods section of the body of the article, besides starting unusually with “Ethical Considerations” it was stated as “All participants who contributed data for analysis provided written informed consent for study participation and publication of the results. Parents or any care giver consented on behalf of study participants who were aged less than 18 years." It needs further clarity for better sense and feasibility on how the secondary data from HDSS/five years or older/can be conducted with obtained written consent from each participant or care giver.

 If possible, it is good to get further confirmation from a biostatistician.

 Unable to assess the availability of all the source data underlying the results **.**

**Approved with Reservations:** The reviewer believes the article has an academic merit. Nonetheless, I think it needs some revisions for better sense and clarity specifically on clearly providing the epidemiological study design employed, and the controversy between using secondary data and obtaining written consent from each participant.

 Furthermore, the data seems obsolete or 5-10 years old/comparing 2012 with that of 2017 in 2022; may need strong justifications for using the research findings to the required interventions timely to solve the observed gap or problem.

Is the work clearly and accurately presented and does it cite the current literature?

Partly

If applicable, is the statistical analysis and its interpretation appropriate?

I cannot comment. A qualified statistician is required.

Are all the source data underlying the results available to ensure full reproducibility?

No

Is the study design appropriate and is the work technically sound?

Partly

Are the conclusions drawn adequately supported by the results?

Yes

Are sufficient details of methods and analysis provided to allow replication by others?

Partly

Reviewer Expertise:

I am a Public Health Expert

I confirm that I have read this submission and believe that I have an appropriate level of expertise to confirm that it is of an acceptable scientific standard, however I have significant reservations, as outlined above.
